# Rapid shear stress-dependent ENaC membrane insertion is mediated by the endothelial glycocalyx and the mineralocorticoid receptor

**DOI:** 10.1007/s00018-022-04260-y

**Published:** 2022-04-10

**Authors:** Zülfü C. Cosgun, Magdalena Sternak, Benedikt Fels, Anna Bar, Grzegorz Kwiatkowski, Marta Z. Pacia, Lisbeth Herrnböck, Martina Lindemann, Johannes Stegbauer, Sascha Höges, Stefan Chlopicki, Kristina Kusche-Vihrog

**Affiliations:** 1 Institute of Physiology II, University of Münster, 48149 Münster, Germany; 2 Jagiellonian Centre for Experimental Therapeutics (JCET), Jagiellonian University, 30-348 Krakow, Poland; 3 Institute of Physiology, University of Luebeck, 23562 Lübeck, Germany; 4 Department of Nephrology, Medical Faculty, University Hospital Düsseldorf, Heinrich-Heine-University Düsseldorf, Düsseldorf, Germany; 5 Chair of Pharmacology, Medical College, Jagiellonian University, Kraków, Poland; 6 DZHK (German Research Centre for Cardiovascular Research), Partner Site Hamburg/Kiel/Lübeck, Lübeck, Germany

**Keywords:** ENaC, Mineralocorticoid receptor, Glycocalyx, Shear stress, Endothelial dysfunction

## Abstract

The contribution of the shear stress-sensitive epithelial Na^+^ channel (ENaC) to the mechanical properties of the endothelial cell surface under (patho)physiological conditions is unclear. This issue was addressed in in vivo and in vitro models for endothelial dysfunction. Cultured human umbilical vein endothelial cells (HUVEC) were exposed to laminar (LSS) or non-laminar shear stress (NLSS). ENaC membrane insertion was quantified using Quantum-dot-based immunofluorescence staining and the mechanical properties of the cell surface were probed with the Atomic Force Microscope (AFM) in vitro and ex vivo in isolated aortae of C57BL/6 and ApoE/LDLR^-/-^ mice. Flow- and acetylcholine-mediated vasodilation was measured in vivo using magnetic resonance imaging. Acute LSS led to a rapid mineralocorticoid receptor (MR)-dependent membrane insertion of ENaC and subsequent stiffening of the endothelial cortex caused by actin polymerization. Of note, NLSS stress further augmented the cortical stiffness of the cells. These effects strongly depend on the presence of the endothelial glycocalyx (eGC) and could be prevented by functional inhibition of ENaC and MR in vitro endothelial cells and ex vivo endothelial cells derived from C57BL/6, but not ApoE/LDLR^-/-^ vessel. In vivo In C57BL/6 vessels, ENaC- and MR inhibition blunted flow- and acetylcholine-mediated vasodilation, while in the dysfunctional ApoE/LDLR^-/-^ vessels, this effect was absent. In conclusion, under physiological conditions, endothelial ENaC, together with the glycocalyx, was identified as an important shear stress sensor and mediator of endothelium-dependent vasodilation. In contrast, in pathophysiological conditions, ENaC-mediated mechanotransduction and endothelium-dependent vasodilation were lost, contributing to sustained endothelial stiffening and dysfunction.

## Introduction

Healthy endothelium is defined by its anti-thrombotic and anti-inflammatory activity, barrier function, modulation of vessel tone and blood pressure. In contrast, dysfunctional endothelium displays pro-thrombotic and pro-inflammatory phenotype and is causally responsible for the development of atherosclerosis and other cardiovascular disorders [1–3], while some drugs reversing endothelial dysfunction prevent cardiovascular diseases [4, 5]

Endothelial function is modulated by the shear stress of the streaming blood [6]. Due to their strategic position in the blood vessel, endothelial cells generally sense and react to changes in shear stress which is physically defined as the tangential force derived by the friction of the flowing blood on the endothelial surface. It is the product of the shear rate at the wall and the blood viscosity. Important elements of the shear stress sensing machinery are represented by the flow-sensitive glycocalyx endothelial (eGC) on top of endothelial cells, plasma membrane inserted mechano-sensitive ion channels, and the cortical actin-rich network located 50–150 nm underneath the plasma membrane. Thus, shear stress- and mechanotransduction by endothelial cells are dependent on the faultless interaction of these elements. Recently, the mechano-sensitive epithelial Na^+^ channel (ENaC) was identified as an important shear stress sensor. Knoepp et al. reported that glycosylated asparagines in the palm and knuckle domains of the ENaC α-subunit are important for shear stress sensing [7]. In accordance with the force-from-filament principle, these structures may provide a connection to the extracellular matrix (ECM), providing a complex flow sensing structure at the cellular surface [7].

ENaC is known to be mechano-sensitive and its activity (P_o_) can be increased by laminar shear stress [8–12]. Similar to the epithelial ENaC, endothelial ENaC can also sense and be regulated by the blood flow‐induced shear stress [13].

In addition to mechano-sensitive ion channels, the mechanical properties (i.e., cortical stiffness) of the endothelial surface itself have a crucial impact on shear stress sensing and transmission of the signal into the interior of the cell [14]. The continuous change between a ‘soft’ and ‘stiff’ condition of endothelial cells is physiologically important and guarantees flexible adaptations to changes in blood pressure or shear stress levels maintaining vascular homeostasis. In contrast, persistent stiffening of the endothelial cortex can be seen as a pathophysiological condition and was recently defined as ‘stiff endothelial cell syndrome’ (SECS) [15] and was linked to dysfunctional endothelium. Aldosterone, a high salt load or pro-inflammatory substances for example, were shown to contribute to the development of SECS [16–20]. The mechanical properties of the endothelial surface in turn determine the nitric oxide (NO) production and maintain flow-mediated vasodilation (FMD). Of note, FMD represents a widely accepted method to assess endothelial function in humans that has a prognostic value in cardiovascular disease in humans [21].

Recently, the mechano-sensitive endothelial ENaC was suggested to be a major regulator of the endothelial mechanics and vascular function [22, 23]. This is based on the fact that ENaC-dependent changes in the cortical stiffness-degree affect the release of NO from the endothelial cells, as cortical stiffness and NO release are inversely correlated [24]. Moreover, the endothelial NO-synthase (eNOS) is co-localized with cortical F-actin in the plasma membrane of endothelial cells and it could be shown that polymerization of the actin cytoskeleton (shift from G- to F-actin) decreases eNOS activity/NO release [25] and also vice versa [26]. Pharmacological (benzamil, amiloride or spironolactone) as well as genetic (siRNA and endothelial αENaC KO mice) manipulation of ENaC function/expression, affect the cortical stiffness [24, 27]. It was shown that a reduced ENaC membrane abundance and/or activity modulates the bioavailability of NO, linking endothelial nanomechanics with endothelial function [16, 28].

Recently, a correlation between ENaC-dependent endothelial plasticity (i.e., the ability to flexibly adapt the stiffness-degree) and an increased pulse wave velocity (PWV) was found in a patient cohort [29]. This indicates that endothelial function correlates with overall arterial stiffness, but the underlying mechanisms are insufficiently understood.

There is given consent that (i) ENaC acts as a shear stress sensor, (ii) ENaC activity is dependent on flow and (iii) ENaC function maintains vascular responsiveness. However, little is known about the underlying mechanisms, especially in the comparison of physiological and pathophysiological conditions.

To better understand a possible distinct role of ENaC-dependent mechanosensing in the regulation of endothelial function, we studied ENaC-dependent mechanisms under flow condition in in vitro microfluidic model mimicking laminar and non-laminar shear stress conditions as well as in vivo in control-wild-type mice and in ApoE/LDLR^-/-^mouse model for endothelial dysfunction and atherosclerosis. Our data suggest a cross-talk between ENaC-dependent endothelial mechanosignalling, hemodynamic forces and vascular function that is preserved in healthy mice but is lost in the setting of endothelial dysfunction in mice with atherosclerosis. Thus, following the concept of mechanomedicine [30], we identified the endothelial surface as an important hub for the regulation of vascular function.

## Methods

### Cell and tissue culture

Human umbilical vein endothelial cells (HUVEC) were isolated as described previously [24]. Cells were seeded on fibronectin-coated glass coverslips or microslides (µ-Slide I, 0.6 Luer, IBIDI GmbH, Gräfeling, Germany) and cultivated in Medium 199 (GIBCO, Invitrogen Corp., Karlsruhe, Germany) supplemented with penicillin G, streptomycin, heparin, FCS (fetal calf serum), and retinal growth factor, freshly extracted from calf eyes, until confluence was reached (3–5 days). Experiments were conducted on monolayers of the second or third passage. Twenty-four hours prior to the experiments, aldosterone (1 nmol/L) was added to the culture medium. In the subsequent experiments, substances were used as follows: 100 nM canrenoate was acutely applied to the medium while the 15 min application of shear stress. Brefeldin A (BFA, 5 µg/ml) was added 90 min and Heparinase I (1 SU) was applied 60 min prior to shear stress experiments.

### Animals, aorta preparation and cultivation

Aortae derived from ApoE/LDLR^-/-^ (apolipoprotein E-deficient and Low-Density Lipoprotein Receptor-Deficient Double Knockout mouse, bred in the Department of Human Nutrition, University of Agriculture in Krakow, Poland) and C57/BL/6 (WT, (from Mossakowski Medical Research Centre, Polish Academy of Sciences, Warsaw, Poland)) mice at the age of 3–6 months were prepared for the experiments as described elsewhere [18, 24]. All mice (body weight of 20–30 g) were bred in standard conditions (LD: 12/12, humidity: 60%, temperature: 23 °C), and housed in pathogen-free settings. In brief, animals were euthanized by an intraperitoneal injection consisting of ketamine (100 mg per kg body weight; Vetoquinol Biowet, Poland) and xylazine (10 mg per kg body weight¸ Sigma Aldrich, München, Germany). In the case of stiffness measurements with the Atomic Force Microscope (AFM) [31] and immunofluorescence stainings, the thoracic aorta was isolated and stored in a modified version of 4 °C chilled solution 8 [24]. Cleaning of connective tissue under microscopic visualization and further preparations were conducted within 1–2 days as recently described [24]. The procedure results in aortic preparations with the endothelial surface facing upwards, thus enabling in situ experiments on living endothelial cells as described before [24]. After preparation, the in situ patches were cultured at 37 °C and 5% CO_2_ in minimal essential medium (MEM, GE Healthcare, Austria), containing 20% bovine fetal serum (PAA, Austria), 1% MEM vitamins (Gibco, UK), penicillin (10.000 U/ml, Biochrom, Germany), streptomycin (10.000 µg/ml, Biochrom, Germany) and 1% MEM non-essential amino acids (MEM NEAA, Gibco, UK) for 24–48 h. Depending on group allocation, either ethanol (Sigma Aldrich) or 100 nM spironolactone (solvent control, Sigma Aldrich) was added to the medium.

### Shear stress experiments

For flow-exposure experiments, shear stress was applied to HUVECs using a conventional microfluidic system (IBIDI). Cells were mounted on fibronectin-coated microslides (µ-Slide I, 0.6 Luer, IBIDI) and incubated at 37 °C with 5% CO_2_. After 30 min of sedimentation, the cells were grown for 48 h before application of shear stress. The medium was changed on a daily basis. Once cells attained confluency, culture media was allowed to pass through cells using the IBIDI pump system to apply unidirectional non-pulsatile shear stress of 8 dyn/cm^2^ on the HUVECs for a period of either 48 h for chronic effects or 15 min for acute effects. Initially, the pressure was gradually increased from 2 to 8 dyne/cm^2^. For static experiments, cells were seeded on μ slide, and no shear stress was applied.

To apply non-laminar shear stress, y-shaped microslides (µ-Slide y-shaped, IBIDI) were employed under the same conditions. With this in vitro microfluidic system, it is possible to simulate blood vessel bifurcations and thus the impact of inhomogeneous shear stress. Non-laminar shear stress can be monitored in bifurcations of the channel Thus, this system represents a reliable in vitro model for atherosclerotic processes. Substances were added as appropriate directly into the pump system.

### Immunofluorescence staining

To study the membrane abundance of ENaC, immunofluorescence stainings were performed as described elsewhere [24, 32]. To avoid differences in cell shape and density of HUVEC, the culture conditions were strictly controlled and only confluent cells were used for the experiments. HUVECs and in situ endothelial cells derived from ex vivo aorta preparations were fixed with 0.1% glutaraldehyde and gently washed four to five times in phosphate-buffered saline (PBS, in mmol/L: 140 NaCl, 2 KCl, 4 Na_2_HPO_4_, 1 KH_2_PO_4_, pH 7.4) at room temperature. Then, the in situ endothelial cells were washed again in PBS and blocked with 10% normal goat serum (NGS) at room temperature for 30 min to block unspecific binding sites. As described previously, a specific polyclonal anti-αENaC antibody was used in a 1:250 dilution (Santa Cruz Biotechnology, USA) [33]. For QuantumDot (QD) labeling, cells were incubated with QD655goat F(ab’)2 anti-rabbit IgG conjugates (1:800) (QuantumDot, Hayward, USA).

For the heparan sulfate (HS) stainings, a specific Anti-HS (10E4 epitope) mouse antibody (AMS Biotechnology, Abingdon, UK) and as a secondary antibody QD565goat F(ab’)_2_ anti-mouse IgG conjugates (QuantumDot) were used. As a negative control, cells were stained only with the secondary antibody (QD655 or QD565).

Staining was verified by epifluorescence microscopy (microscope: Leica DMI 6000B, Leica Microsystems; camera: CoolSNAPHQ, Photometrics). The QD-based immunofluorescence was quantified by counting QD/1000 µm^2^ of cell surface area using ImageJ software (National Institutes of Health, USA). Images were taken in 3 different Z-sections of the endothelial monolayer and all images were analyzed simultaneously with identical exposure times to account for any variations in cell height or staining intensities. QD background levels (QD detected in negative controls) were subtracted from the results. The quantified ENaC/QD number per area was normalized to the respective control group for further analysis.

For staining of cortical F-actin, HUVEC and endothelial cells derived from ex vivo aorta preparations were fixed with 4% formaldehyde for 30 min and gently washed with PBS afterwards. For permeabilization purposes, Triton X-100 (Sigma Aldrich, Germany) was added to the cells for 1 min. Phalloidin–Tetramethylrhodamine B isothiocyanate (Phalloidin-TRITC, Sigma Aldrich) was added in a dilution of 1:100 for 60 min. The cells ex vivo patches were then mounted on microscopy slides with medium containing 4′,6-diamidino-2-phenylindole (DAPI). The fluorescence images were conducted with an inverted confocal microscope (TCS SP8, Leica Microsystems, Wetzlar, Germany) equipped with a 63 × NA 1.4 objective. Z stacks of the entire cell or the apical part were recorded and later visualized by maximum intensity projection [34].

### Atomic force microscopy measurements

Mechanical stiffness of the endothelial cortex was determined using an atomic force microscope (AFM; MultiMode SPM, Bruker, Berlin, Germany) as described elsewhere [24]. Briefly, a MultiMode 3 SPM AFM (Bruker, Germany) equipped with a feedback-controlled heating device (Nanoscope Heater Controller; Digital Instruments, Veeco, USA) was used to create force–distance-curves (FDC) of the endothelial cells in vitro and ex vivo, which were prepared and cultured as described above. To determine exclusively the stiffness of the endothelial cell cortex, soft triangular cantilevers (Novascan, USA), with a nominal spring constant of 0.03 N/m and a polystyrene sphere (10 µm) as a tip, were used, whereas a ramp size of 2 µm and a trigger threshold of 100 nm were chosen as described elsewhere [24]. Measurements were performed at 37 °C in HEPES-buffered solution (composition in mmol/l: NaCl 140, KCl 5, MgCl_2_ 1, CaCl_2_ 1, glucose 5, HEPES 10 (N-2-hydroxyethylpiperazine-N’-2-ethanesulfonic acid), pH 7.4). To ensure an intact endothelial function, 1% FCS was added to the buffer during the measurements. According to the pre-incubation of the ex vivo preparations (WT and ApoE/LDLR^-/-^), either ethanol, 100 nM spironolactone (chronic, 24 h) or 1 µM amiloride (acute, 30 min) was added to the buffer during the measurements. From each preparation, the stiffness of approximately 20 endothelial cells was determined, whereas 6 FDC of each cell were taken and averaged.

To study the shear stress-induced changes of the mechanical properties of endothelial cells, HUVEC grown on microslides were fixed with 0.1% glutaraldehyde immediately after application of shear stress with the IBIDI pump system. Then the polymer coverslip, at the bottom of the microslides carrying the cells, was manually cut out (about 1 mm^2^) and mounted on a glass coverslip using CellTak similar to the procedure described for aortic ex vivo patches [24]. Stiffness measurements of fixed endothelial cells were also conducted with the MultiMode 3 AFM as described elsewhere [35]. During the measurements, endothelial cells were constantly bathed in phosphate-buffered saline (PBS).

All obtained AFM data were collected with NanoScope software 5.31 and V8.10 (Bruker). Stiffness values were calculated from FDC using the Protein Unfolding and Nano-Indentation Analysis Software PUNIAS 3D version 1.0 release 2.2 (http://punias.voila.net).

### MRI-based assessment of endothelial function in vivo

4–5-month-old ApoE/LDLR^-/-^ mice and age-matched control C57BL/6 mice were anaesthetized using isoflurane (Aerrane, Baxter Sp. z o. o., Poland, 1.7 vol%) in oxygen and air (1:2) mixture and imaged using a 9.4 T scanner dedicated for magnetic resonance imaging (MRI) of mice (BioSpec 94 USR, Bruker, Germany), in the supine position. The activity of the heart, respiration and body temperature (maintained at 37 °C using circulating warm water) were monitored using a Monitoring and Gating System (SA Instruments Inc., Stony Brook, NY, USA). All experiments were approved by the Ethics Local Committee of Jagiellonian University (Krakow, Poland) and were compliant with the Guide for the Care and Use of Laboratory Animals of the National Academy of Sciences (NIH publication No. 85–23, revised 1996).

Endothelium-dependent vascular responses in vivo were assessed by 2 techniques, as described previously in a number of studies [36–41], measurements of endothelium-dependent response to acetylcholine (Ach) and flow-mediated dilation (FMD) in response to reactive hyperemia, the latter considered to be a gold standard in studies on endothelial dysfunction in humans [42]. Response to injection of Ach (Sigma–Aldrich, Poznan, Poland: 50 μl, 16.6 mg/kg, *i.p*.), was analyzed in the abdominal (AA) and thoracic (TA) parts of aorta. The vasomotor responses were examined by comparing two, time-resolved 3D images of the vessels prior to and 25 min after intraperitoneal Ach administration (time was determined experimentally in our previous study [37] and the dose of Ach used to assess endothelium-dependent vasodilation in vivo in mice was based on previous study [43]. FMD after short-term occlusion was assessed in the femoral artery (FA). Artery occlusion was induced by a home-made vessel occluder, described elsewhere [39]. Briefly, vessel occluder was placed in measurement bed while loop was put on mice hind limb. At the beginning of the experiment, 3D image of femoral artery was obtained before vessel occlusion and then mice were pulled out from MRI scanner to provide proper loop positioning. Vessel occlusion was induced by pulling of the syringe plunger resulting in the loop closing. In the next step, positioning measurements were repeated, and after 5 min of vessel occlusion [44], the clamp was released by pushing the syringe plunger. 3D image of the femoral artery after vessel occlusion was obtained immediately after loop release.

The MRI-based methodology allowed us to measure endothelial function in physiological flow conditions in vivo based on the magnitude of endothelium-dependent vasodilation in the large vessels (the aortae and femoral artery) in response to chemical stimulation (acetylcholine in the aorta) or in response to increased flow induced by 5 min vessel occlusion in the FA (flow-mediated vasodilation). Previously, this methodology was well validated and repeatedly used in our studies in various murine models of cardiovascular diseases in vivo [36–41, 45, 46].

FMD and Ach-induced responses were measured 30 min and 1 h after benzamil (1 mg/kg, *i.v*) or canrenone (10 mg/kg, *i.v*) administration, respectively.

Images were acquired using the cine IntraGate™ FLASH 3D sequence, reconstructed with the IntraGate 1.2.b.2 macro (Bruker). Volumes of the vessels were analyzed using ImageJ software 1.46r (NIH Bethesda, Maryland, USA) using scripts written in Matlab (MathWorks, Natick, MA, USA), in the hyperstack (3D images of AA were positioned on the sagittal view of the mice) of the AA (10 slices in diastole, from the renal arteries down) and the TA (10 slices in diastole, from the celiac artery up) or in the hyperstack (3D images of FA were positioned on the coronal view of the mice, on the right hind limb of the mouse) of the FA (7 slices). All cross-sectional areas of vessels at each slice were obtained using thresholding segmentation and exported to Matlab, where vessel volumes were reconstructed and calculated. Imaging parameters included the following: repetition time (TR)—6.4 ms, echo time (TE)—1.4 ms, field of view (FOV)—30 × 30x5 mm^3^ for the FA and 30 × 30x14 mm^3^ for the aorta, matrix size—256 × 256x30 for the FA and 256 × 256x35 for the aorta, flip angle—30°, and the number of accumulations (NA)—15. Total scan time was 10–12 min.

### Statistical analysis

The significance of the difference in each data set was determined using 1-way ANOVA, 2-way ANOVA with Tukey post hoc test or Kruskal–Wallis–ANOVA depending on a normal distribution. When significant differences between groups were detected, post hoc comparisons were made by Student’s *t* test (parametric) or Mann–Whitney-U test (non-parametric). Asterisks refer to: *p ≤ 0.05; **p ≤ 0.01, ***p ≤ 0.001 or respective lower p-values. All specified data were calculated as median (Q_1_: 1st quartile, Q3: 3rd quartile) and are displayed as box plots: mean (square), median (horizontal line), 25th and 75th percentile, outlier (whiskers). Specified n-numbers refer to cells analyzed, whereas N-numbers refer to independent experiments. For AFM measurements, *N* = number of mice, *n* = number of single measured endothelial cells per aorta.

## Results

### Increase of ENaC membrane abundance by acute and chronic shear stress: the role of glycocalyx and MR

**Fig. 1:**
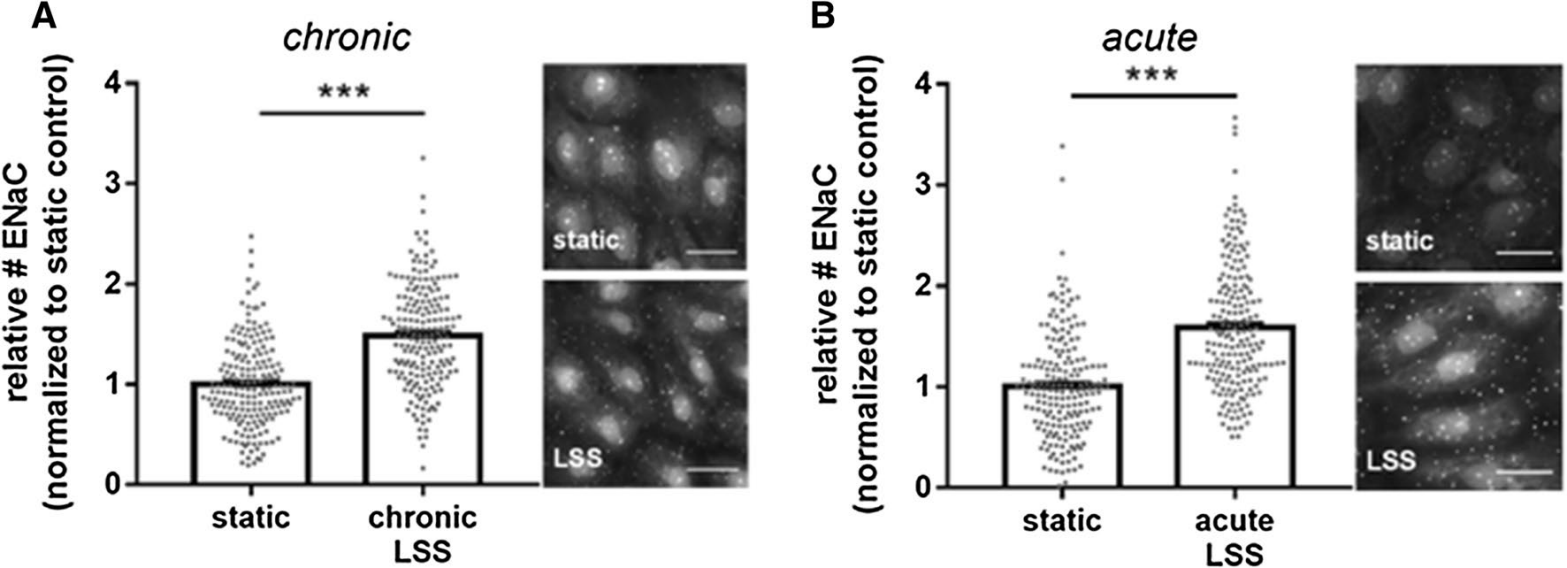
Shear stress-induced ENaC membrane abundance within minutes. **A** ENaC membrane abundance was quantified under chronic laminar shear stress (48 h, 8 dyne/cm^2^) and showed a significant increase compared to non-flow controls by 48.6 ± 3.6%. **B** Application of acute (15 min) laminar shear stress (8 dyne/cm^2^) results in an increase of ENaC expression by 58.5 ± 4.5%. 1 nM aldosterone was present in both conditions (*N* = 3; *n* = 195−198; *** = significant difference (*p* ≤ 0.001)). Scale bar: 25 μm

**Fig. 2:**
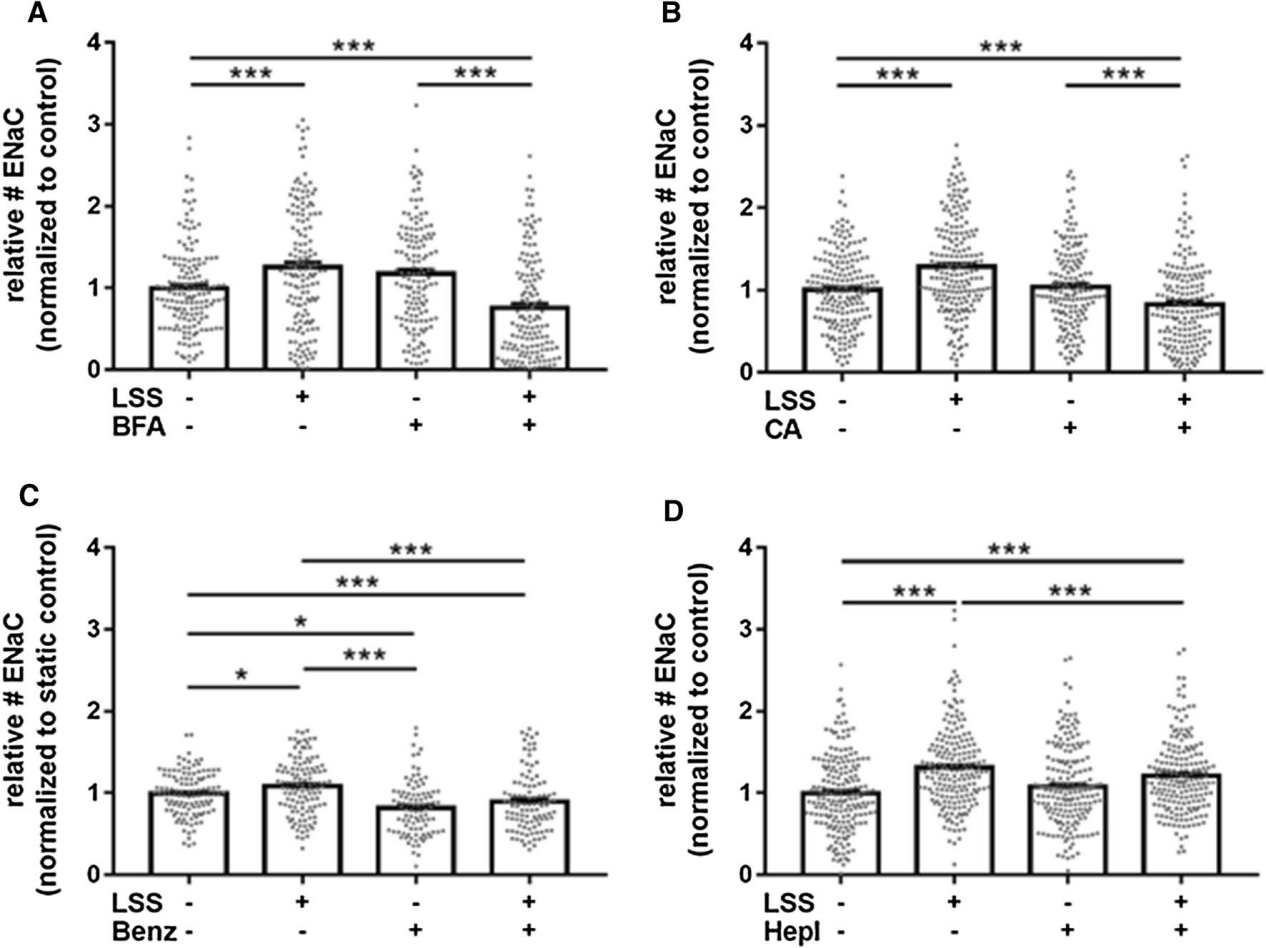
Analysis of αENaC membrane expression. **A** Under acute (15 min) LSS (8 dyne/cm^2^), application of Brefeldin A (5 μg/ml) reduced the membrane abundance of ENaC by 32.3 ± 5.2% (*N* = 3, *n* = 142). These observations indicate a non-genomic membrane insertion of ENaC. **B** Application of CA (100 nM), the active metabolite of the MR antagonist spironolactone and/or acute (15 min) application of LSS (8 dyne/cm^2^) prevents the LSS effect under flow conditions by 20.7 ± 4.0% compared to static control conditions (*N* = 3, *n* = 157). **C** Application of Benzamil (1 µM) prevents the LSS effect under flow conditions by 20.7 ± 4.0% compared to static control conditions (*N* = 3, *n* = 117). **D** Enzymatic removal of the glycocalyx with heparinase I (1 SU/ml) reduced the ENaC membrane abundance only under LSS conditions 13 ± 3.0% (*N* = 3, *n* = 95) and was still augmented compared to static conditions by 21.1 ± 3.2% (*N* = 3, *n* = 195), whereas under static conditions no effect could be observed (* = significant difference (*p* ≤ 0.05), *** = significant difference (*p* ≤ 0.001))

The regulation of ENaC by shear stress was recently reported [8–12], but the underlying mechanisms and the time course are unclear up to now. To study the basic effects of physiological relevant shear stress (8 dyne/cm^2^) on the membrane insertion and abundance of ENaC, LSS was chronically applied for 48 h using the IBIDI pump system. Immunofluorescence-based quantification of ENaC molecules in the membrane of fixed, non-permeabilized cells revealed that chronic shear stress significantly increased ENaC membrane abundance by 48.6 ± 3.6% (*N* = 3, *n* = 195, *p* ≤ 0.01) compared to controls without LSS (Fig. 1A). In particular, to test whether acute shear stress activates ENaC, LSS (8 dyne/cm^2^) was applied for 15 min to an endothelial cell monolayer and the ENaC membrane abundance was again quantified using a QD-mediated immunofluorescence approach. It was found that acute LSS augmented the ENaC membrane abundance by 58.5 ± 4.5% compared to static conditions (*N* = 3, *n* = 195, *p* ≤ 0.01) (Fig. 1B), indicating the rapid membrane insertion of preformed ENaC molecules which might be localized in vesicles beneath the plasma membrane [47, 48]. To test the involvement of vesicular transport of ENaC between the endoplasmatic reticulum and the golgi apparatus in ENaC membrane insertion, Brefeldin A (BFA) was applied. As shown in Fig. 2A, BFA prevented the acute effects of shear stress, resulting in a significantly lower amount of ENaC in the plasma membrane (*N* = 3, *n* = 141–157, 90 min pre-incubation) supporting the role of vesicle transport and intracellular trafficking in ENaC membrane insertion in response to LSS. Importantly, application of the aldosterone receptor antagonist canrenone (CA), the active metabolite of the mineralocorticoid antagonist spironolactone, also prevented the rapid shear stress-induced ENaC membrane insertion in the presence of aldosterone (*N* = 3, *n* = 157–194, *p* ≤ 0.01) (Fig. 2B). This indicates that the MR-dependent mechanism is also involved in the rapid membrane insertion of ENaC. Furthermore, functional inhibition of ENaC using benzamil reduced the number of shear stress-induced channels in the endothelial plasma membrane (Fig. 2C) which is in agreement with previous findings [32].

Given the fact that the eGC was identified as an important shear stress sensor in vascular endothelial cells [49] and to test the role of the eGC in the context of ENaC regulation by shear stress, heparinase I (HepI) was employed to enzymatically remove the heparan sulfates (HS) as they represent the major parts of the eGC. Removal of the mechanosensing eGC led to a significant decrease in ENaC membrane abundance under shear stress conditions by 13.0 ± 3.0% (*N* = 3, *n* = 195, *p* ≤ 0.01), whereas under static conditions, no effect was observed (Fig. 2D). This indicates that the presence and integrity of the eGC are important for signal transduction of shear stress into the cell regulating rapid ENaC membrane insertion.

### Non-laminar shear stress induces actin polymerization and increases cortical stiffness

Blood flow-induced shear stress is an essential feature in the development of atherosclerosis. In particular, non-laminar shear stress (NLSS) can be seen as a promotor of atherogenesis. To test whether acute NLSS influences the nanomechanical properties and function of endothelial cells and thus contributes to the development of endothelial dysfunction, specific y-shaped cell culture slides (IBIDI) were employed to mimic conditions with disturbed blood flow that occurs in atherosclerosis.

**Fig. 3:**
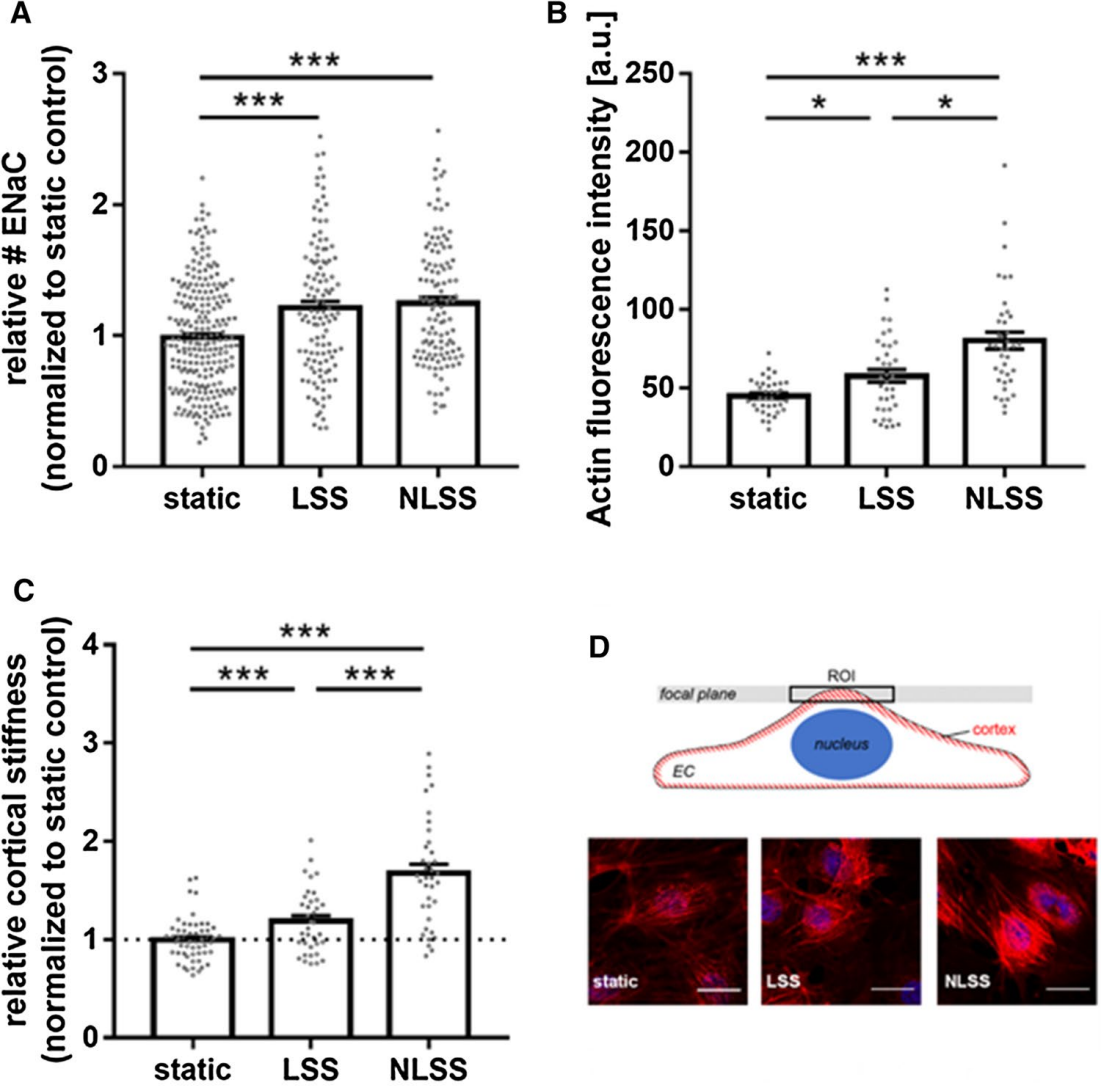
Impact of non-laminar shear stress. **A** ENaC membrane abundance was quantified and showed a significant increase in LSS (8 dyne/cm^2^) conditions compared to non-flow controls by 21.4 ± 4.7%. NLSS (8 dyne/cm^2^) increased ENaC membrane abundance as well (+ 25.0 ± 4.3%), but it did not matter which form of shear stress was applied (*N* = 3, *n* = 120). **B** LSS lead to stiffening of the cell cortex by 18.9 ± 5.5% compared to static controls. NLSS further increased the cortical stiffness by 67.9 ± 8.9% compared to non-flow conditions (*N* = 3, *n* = 37) (* = significant difference (*p* ≤ 0.05, *** = significant difference (*p* ≤ 0.001)). **C** Cortical F-actin was quantified by confocal microscopy and showed a significant increase by 19.2 ± 4.0% in LSS conditions compared to non-flow controls. NLSS further increased F-actin by 65.5 ± 5.4% compared to static controls (*N* = 3, *n* = 39). **D** Scheme and representative images of the analysis of cortical actin. Static, LSS and NLSS conditions are shown

In this model, it was demonstrated that acute application of NLSS increased ENaC membrane abundance compared to static conditions (+ 21.4 ± 4.7%, *N* = 3, static *n* = 237, LSS/NLSS *n* = 120, *p* ≤ 0.01), but had no additional effect compared to LSS (Fig. 3A).

Reorganization of the cortical actin cytoskeleton was shown to significantly contribute to changes in endothelial nanomechanics and function [25, 34]. Accordingly, application of LSS significantly enhanced the presence of cortical F-actin compared to static conditions by 19.2 ± 4.0% (*N* = 3, *n* = 36, *p* ≤ 0.01). Of note, NLSS further increased the amount of cortical F-actin (65.5 ± 5.4%, *N* = 3, *n* = 39, *p* ≤ 0.01) compared to static controls (Fig. 3B).

Using AFM-based nanoindentation measurements, we could link shear stress-induced ENaC membrane abundance and actin polymerization to the nanomechanical properties of the endothelial cortex in that LSS led to stiffening of the cell cortex by 18.9 ± 5.5% (*N* = 3, *n* = 37, *p* ≤ 0.01) compared to static controls which can be explained by an enhanced amount of F-actin under LSS condition. Importantly, the application of NLSS further increased the cortical stiffness by 67.9 ± 8.9% (*N* = 3, *n* = 36, *p* ≤ 0.01) (Fig. 3C) paralleled by further, NLSS-induced actin polymerization. These results underscored the effects of NLLS in a reliable in vitro model. Figure 3D shows exemplary confocal images of cortical actin, without effects on stress fibers.

### Distinct effects of ENaC or MR inhibition on endothelial nanomechanics in C57BL/6 as compared with ApoE/LDLR^-/-^ mice: an ex vivo approach

The contribution of ENaC to endothelial nanomechanics in a pathophysiological condition was studied in the ApoE/LDLR^-/-^ mouse model for endothelial dysfunction and compared with control C57BL/6 mice (WT).

**Fig. 4:**
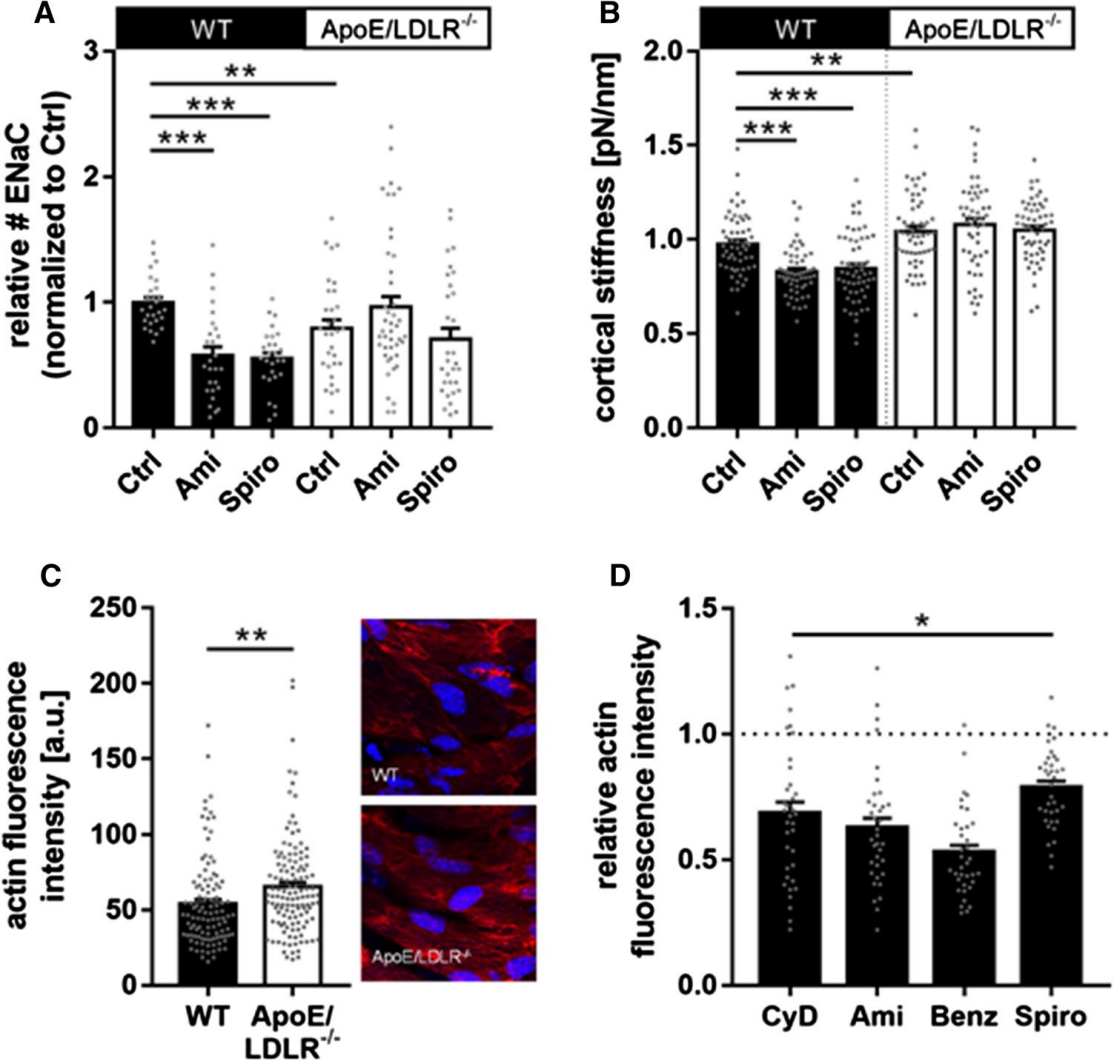
Changed ENaC regulation and endothelial mechanics in ApoE/LDLR^-/-^ ex vivo endothelial cells. **A** In ApoE/LDLR^-/-^ ex vivo endothelial cells, the ENaC membrane expression is reduced compared to WT by − 23%. In WT, amiloride and spironolactone significantly decreased the ENaC membrane expression by − 43% and − 41% (*N* = 3, *n* = 28), respectively. In the ApoE/LDLR^-/-^ mouse model, this effect is abolished, indicated an alternative ENaC regulation under dysfunctional conditions. Compared to WT, in ApoE/LDLR^-/-^ ex vivo endothelial cells the ENaC membrane expression is not reduced after application of amiloride and spironolactone (*N* = 3, *n = *32–44, **significant difference = *p* ≤ 0.01, ***p ≤ 0.001). **B** The cortical stiffness of endothelial cells ex vivo derived from ApoE/LDLR^-/-^ aortae is increased compared to WT by 6% (*N* = 3; WT: 0.98 ± 0.021 pN/nm; ApoE/LDLR^-/-^: 1.04 ± 0,025 pN/nm). In WT, amiloride and spironolactone softens the endothelial cortex by − 17% (*N* = 3, *n* = 28) and − 20% (*N* = 3, *n* = 56, ** significant difference = *p* ≤ 0.01, ***p ≤ 0.001), respectively. This effect is absent in the ApoE/LDLR^-/-^ model. **C** Phalloidin stainings revealed the amount of F-actin in ApoE/LDLR^-/-^ endothelial cells is significantly increased to WT by 14% (*N* = 4, *n* = 109–125, ** significant difference = *p* ≤ 0.01). **D** In in vitro endothelial cells, actin is depolymerized by CyD (− 28%), amiloride (− 41%), benzamil (− 49%) and spironolactone (− 18%). (*N* = 3; *n* = 60, * significant difference = *p* ≤ 0.05)

Therefore, ex vivo endothelial cells in the isolated aorta were probed for ENaC membrane abundance in endothelial cells (Fig. 4A), ENaC-dependent mechanical stiffness of the endothelial cortex (Fig. 4B), and the actin polymerization level (Fig. 4C).

QD-based immunofluorescence stainings of αENaC-membrane abundance revealed a decreased αENaC levels in ApoE/LDLR^-/-^ aortic endothelial cells compared to WT (-23%, WT control: *N* = 3, *n* = 28; ApoE/LDLR^-/-^ control: *N* = 3, *n* = 32, *p* ≤ 0.01) (Fig. 4A). As reported before [24, 32], functional ENaC inhibition with amiloride or application of the MR antagonist spironolactone reduced the amount of αENaC in the WT (approx. − 43% as compared to control; control: 100.0 ± 3.79, *N* = 3, *n* = 28; amiloride: 57.19 ± 6.57, *N* = 3, *n* = 28; spironolactone: 56.65 ± 4.65, *N* = 3, *n* = 28, *p* ≤ 0.001) (Fig. 4A). Of note, in the ApoE/LDLR^-/-^ endothelial cells ENaC or MR inhibition has no effect on the ENaC membrane abundance (control: 76.73 ± 7.25, *N* = 3, n = 32; amiloride: 96.04 ± 8.60, *N* = 3, *n* = 44; spironolactone: 68.37 ± 8.71, *N* = 3, *n* = 32) (Fig. 4A). Cortical stiffness was analogously decreased after amiloride and spironolactone in in situ endothelial cells of ex vivo aorta preparations from C57BL/6 mice (20% each; control: 0.98 ± 0.02 pN/nm, *N* = 3, *n* = 56; amiloride: 0.83 ± 0.02 pN/nm, *N* = 3, *n* = 54; spironolactone: 0.85 ± 0.02 pN/nm, *N* = 3, *n* = 57, *p* ≤ 0.001) which is in agreement with previous publication [24], but was without effect in the ApoE/LDLR^-/-^ aortae (control: 1.04 ± 0.03 pN/nm, *N* = 3, *n* = 57; amiloride: 1.08 ± 0.03 pN/nm, *N* = 3, *n* = 52; spironolactone: 1.05 ± 0.02 pN/nm, *N* = 3, *n* = 56) (Fig. 4B). However, under control conditions, the ApoE/LDLR^-/-^ endothelial cells show a significantly increased cortical stiffness compared to C57BL/6 endothelial cells (+ 6%, *p* ≤ 0.01), despite reduced ENaC membrane abundance (Fig. 4B). Thus, this observation might indicate altered nanomechanical properties due to persistent endothelial dysfunction in the aorta from ApoE/LDLR^-/-^ mice. Accordingly, an increased cortical actin polymerization in the ApoE/LDLR^-/-^ endothelial cells could be shown compared to C57BL/6 endothelial cells using Phalloidin-TRITC stainings for F-actin (+ 14.17%; WT: *N* = 4, *n* = 108, mean: 55.89 ± 3.340 arbitrary units; ApoE: *N* = 4, *n* = 125, Mean: 65.12 ± 2.925 arbitrary units, *p* ≤ 0.005) (Fig. 4C).

As illustrated in Fig. 4D, functional (amiloride and benzamil) and transcriptional (spironolactone) ENaC inhibition lead to decreased actin fluorescence-intensity values within the cortical region in vitro (− 42%, − 50%, and − 19%, respectively) (amiloride: 0.57 ± 0.023; benzamil: 0.49 ± 0.021; spironolactone: 0.82 ± 0.02, (*N* = 3, *n* = 59−67, *p* ≤ 0.05)), indicating depolymerization of the cortical F-actin after application of the Na^+^-channel blockers. As positive control, the destabilization agent cytochalasin D was applied, which reduced cortical F-actin fluorescence intensity by − 28% (0.71 ± 0.030) (*N* = 3, *n* = 60, *p* ≤ 0.05) (Fig. 4D).

### Distinct effects of ENaC or MR inhibition on flow- and Ach-dependent vasodilation in C57BL/6 as compared with ApoE/LDLR^-/-^ mice: in vivo approach

**Fig. 5:**
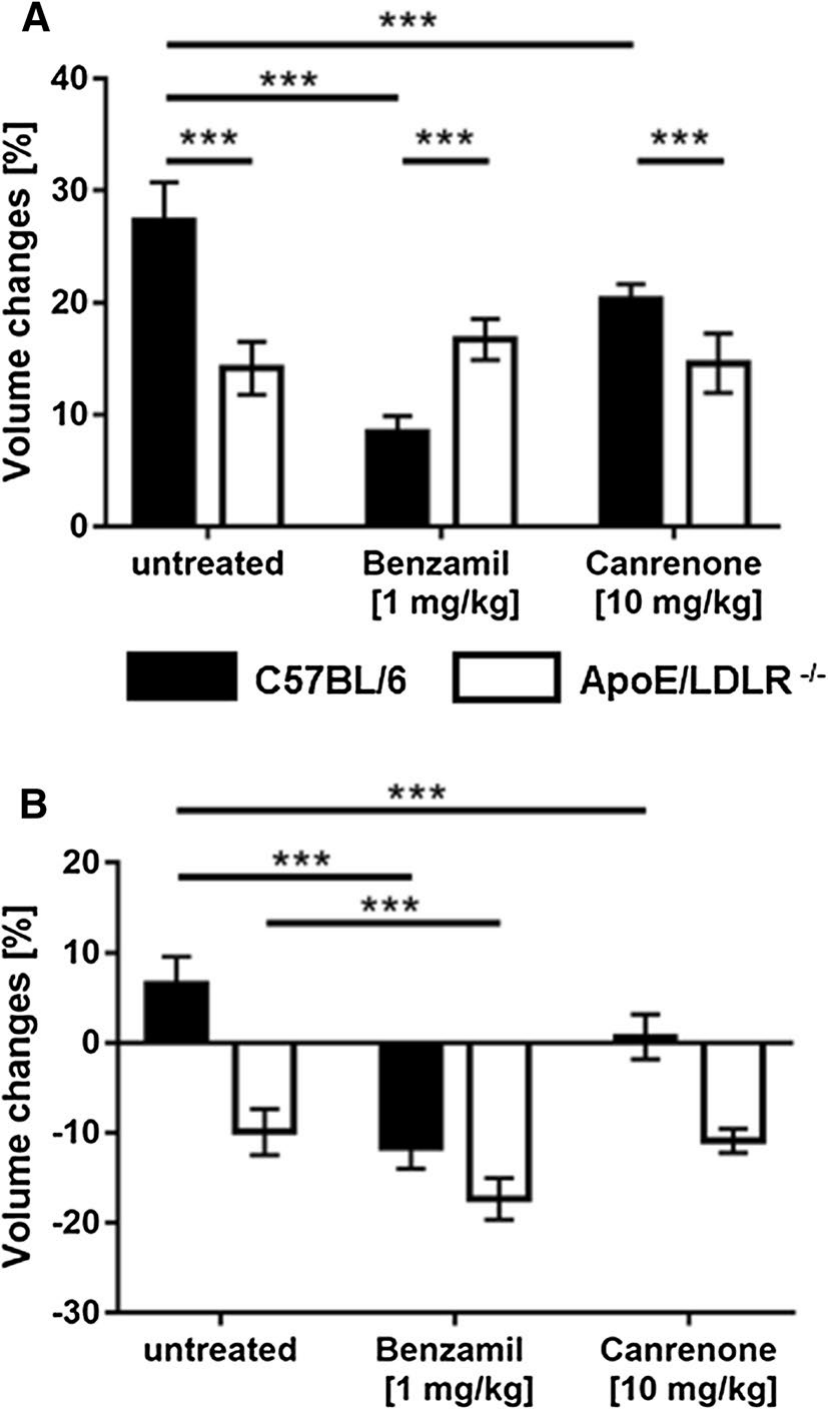
Effects of benzamil and canrenone on endothelial function in vivo in C57BL/6 and ApoE/LDLR^-/-^ mice. **A** Flow-induced (FMD) vasodilation in the femoral artery (FA), after 5 min of vessel occlusion, and acetylcholine(Ach)-induced vasodilation in the abdominal aorta (**B**, AA) 30 min after Ach administration are shown. Results from 4–5-month-old ApoE/LDLR^-/-^ (*n* = 5) and C57BL/6 (*n* = 5) mice pre-treated with benzamil (*n* = 5, both groups) or canrenone (*n* = 5, both groups) in comparison to age-matched, untreated ApoE/LDLR^-/-^ (*n* = 5) and C57BL/6 mice (*n* = 5), respectively. FMD response was measured 30 min after benzamil (1 mg/kg*, i.v*) or canrenone (10 mg/kg, *i.v*) administration. Ach-dependent response was measured 1 h after benzamil or canrenone administration. Statistics: two-way ANOVA followed by Tukey’s post hoc test (normality was assessed using the Shapiro–Wilk test): *** *p* ≤ 0.001

To confirm striking differences in response to ENaC-inhibition in WT *vs*. ApoE/LDLR^-/-^ aortae ex vivo, we tested whether MRI-based assessment of endothelial function in vivo would also reveal distinct effects of ENaC inhibition. We demonstrated that the administration of benzamil or canrenone (CA) prevented the FMD response in the femoral artery (FA) of control C57BL/6 mice (Fig. 5A, volume change of the FA: 8.5% and 20.3%, respectively, vs. 27.3% in untreated mice). In contrast, in ApoE/LDLR^-/-^ mice, displaying impaired FMD, neither benzamil nor canrenone further inhibited FMD (Fig. 5A, 16.7% and 14.6%, respectively, vs. 14.1% in untreated mice).

Moreover, normal Ach-induced vasodilation in C57BL/6 mice, in the abdominal aorta (Fig. 5B, volume change: 6.5%) was completely lost in C57BL/6 mice pre-treated with canrenone or benzamil. In turn, impaired Ach-induced function in ApoE/LDLR^−/−^ mice (− 9.9%) only slightly (without a statistical difference) aggravated in the abdominal aorta (− 17.3%), after pre-treatment with benzamil. Similarly, canrenone pre-treatment of ApoE/LDLR^-/-^ mice, did not modify Ach-induced response in the AA. Altogether, MRI-based assessment of endothelial function in vivo fully recapitulated our findings in ex vivo vessels by showing distinct effects of ENaC or MR inhibition on flow- and Ach-dependent vasodilation in C57BL/6 as compared with ApoE/LDLR^-/-^ mice.

## Discussion

In the present study, it was found that (i) rapid ENaC plasma membrane insertion is regulated by laminar and non-laminar shear stress, (ii) this mechanism is dependent on eGC signaling and the MR and (iii) ENaC regulation is disturbed in the presence of an existing endothelial dysfunction.

Recently, we could correlate ENaC membrane abundance and the degree of cortical stiffness: the more ENaC, the stiffer the endothelial cortex [24]. However, these studies were conducted only under static conditions. Here, we could show that ENaC membrane insertion was stimulated by laminar flow (i.e., LSS, 8 dyne/cm^2^). This supports earlier reports identifying ENaC in epithelial cells or heterologously expressed in oocytes of *Xenopus laevis* as a mechano-sensor, which responds with an increased open probability to higher rates of shear stress [8]. Importantly, a new finding of the present study is that in endothelial cells ENaC membrane insertion under shear stress occurs rapidly within minutes and is sensitive to BFA, indicating that the additionally demanded ENaC molecules are inserted non-genomically from intracellular pools. This rapid ENaC membrane insertion is accompanied by actin polymerization and subsequent mechanical stiffening of the endothelial cortex.

In addition to ENaC, the eGC on top of endothelial cells is recognized as an important shear stress sensor that is anchored via, e.g., syndecan-1 within the cell cortex. This enables efficient transduction of biochemical and biomechanical signals from the intravascular compartment towards endothelial cells, making the eGC a critical interface between the blood and vascular wall [50, 51]. Recently, the importance of a proper eGC could be demonstrated by showing its degradation in an ApoE/LDLR^-/-^ mouse model which could be interpreted as early sign of endothelial dysfunction [38].

Of note, we could demonstrate that under flow conditions, enzymatic removal of the eGC significantly decreased the membrane abundance of ENaC. From these data, we postulate that shear stress sensed by the eGC activates a signaling cascade leading to the rapid non-genomic membrane insertion of ENaC. This is in agreement with a recent study indicating that shear stress may cause deflections of the eGC that in turn is transmitted via N-glycans to αENaC [7]; for review, see [52].

In the present work, we were able to show that in the presence of aldosterone, the rapid shear stress-induced ENaC membrane insertion was mediated by the MR. It is generally accepted that the aldosterone-induced increase in cell surface ENaC expression and channel open probability is regulated in two stages by: (i) trafficking and stabilization of pre-expressed ENaC subunits at the apical cell membrane (non-genomic) and (ii) through the MR-dependent regulation of ENaC subunit gene transcription (genomic) [53]. In agreement with our findings, an aldosterone/MR-dependent non-genomic, rapid membrane insertion of ENaC has been reported in epithelial cells [54, 55], which could be mediated by MR cross-talk, and interaction with different signaling cascades—potentially adding up to a functional synergism between both effects. Recently, a rapid increase in ENaC activity through elevated apical membrane channel density has been linked to vesicle trafficking processes via an intact cortical cytoskeleton and coupled to activation of small GTPases [26, 56–58].

With the rapid membrane insertion of endothelial ENaC due to increased fluid shear stress, the vascular cells possess an effective mechanism to react to hemodynamic changes within minutes. This phenomenon can be regarded as a kidney-independent mechanism of blood pressure regulation and is in line with recent findings showing that endothelial ENaC is indispensable for the regulation of the vascular tone under flow conditions [33].

To mimic atheroprone conditions in vitro, ENaC membrane insertion and the mechanical properties of the endothelial surface were studied under turbulent flow (i.e., NLSS). While the actin polymerization level and cortical stiffness increased, the channel abundance could not be further augmented compared to LSS. This could indicate (i) a saturated expression of the channel under LSS conditions and (ii) a loss of ENaC-responsiveness and/or disturbed signaling under atheroprone conditions (NLSS). It is also likely that turbulent flow leads to an activation of the NF-kappa B pathway, which down-regulates SGK1 expression decreasing ENaC membrane abundance [59]. These observations might explain the contribution of NLSS to pro-atherosclerotic processes—predominantly in branching regions of blood vessels. Previous studies could show a link between the cortical actin cytoskeleton, cortical stiffness and the bioavailability of NO as a marker for endothelial dysfunction [60]. This is supported by data showing that eNOS activity is increased by G-actin [61]—a property of a soft endothelial cell exhibiting a larger G-actin/F-actin ratio and thus possesses a better capacity to release NO during shear stress [62]. The NLSS-induced stiffening is in agreement with data showing that oscillatory flow activates inflammatory pathways, such as NF-κB, contributes to NO-deficiency and promotes atherogenesis. Thus, laminar flow is an important atheroprotective, adaptive process, while regions of arteries under disturbed fluid shear stress are prone to atherosclerosis.

To study the involvement of ENaC-dependent mechanosignaling in the regulation of vascular stiffness, ex vivo endothelial cells in the isolated aorta derived from a mouse model for endothelial dysfunction were employed. In functional, healthy vessels, inhibition of ENaC, either pharmacologically or genetically, resulted in the fall of the channel’s membrane abundance and decreased stiffness of the endothelial cortex, most likely via depolymerisation of the cortical actin [24], which could also be confirmed in this study. As expected, the basal cortical stiffness of ApoE/LDLR^-/-^ endothelial cells was increased compared to WT, indicating a severe functional impairment of the vascular endothelium linked to disturbed endothelial nanomechanics [63]. In agreement with that, we found an increase in cortical endothelial F-actin in ApoE/LDLR^-/-^ ex vivo aorta, indicating a reorganization of the cortical actin-rich mesh. Of note, in the ApoE/LDLR^-/-^ ex vivo endothelial cells in the aorta, the number of membrane ENaC was even lowered compared to WT, which was accompanied by a diminished response to functional ENaC inhibition. This could be caused by the fact that actin assembly and disassembly together with some actin-binding proteins control the membrane insertion and function of ENaC [64]. Thus, the increase in F-actin and cortical stiffness could be responsible for improper ENaC endo- and exocytosis and a loss of functional responsiveness of the channel. Further, it could be speculated that in dysfunctional endothelium, the reduced ENaC membrane abundance is compensatory or the increased cortical stiffness is based on an ENaC-independent mechanism.

From these results, we conclude that under pro-atherogenic conditions the rearrangement of the cortical actin can be seen as the important pathophysiological element of endothelial dysfunction contributing to structural alterations of the cytoarchitecture of the endothelial cells and diminished functional response to ENaC inhibition.

To validate our hypothesis further, we analyze the role of ENaC-dependent mechanosignaling in the regulation of endothelial function by MRI in in vivo conditions with an established protocol [36, 38, 39] in WT and ApoE/LDLR^-/-^ mice. Using this methodology, we demonstrated that ENaC or MR inhibition resulted in blunting of the endothelial-dependent vasodilation in abdominal aortae induced by Ach and in femoral artery induced by flow in C57BL/6 mice. However, in the ApoE/LDLR^-/-^ model for endothelial dysfunction and atherosclerosis [38, 63, 65], ENaC or MR inhibition did not modify these responses that were initially impaired in ApoE/LDLR^-/-^ mice. The contribution of ENaC to endothelium-dependent vasodilation in healthy mice is in agreement with previous findings [33, 39] and supports the notion of the important role of ENaC-dependent mechanosignaling in the regulation of endothelial function. These findings can be explained by the fact that ENaC is an important shear stress sensor and its proper function is imperative to transduce the appropriate signal coming from the streaming blood into vasodilation response [7, 8]. Indeed, endothelium-dependent vasodilation of FMD but also the response induced by Ach in in vivo conditions is obviously modulated by flow [66]. Of note, Ashley et al. demonstrated that ENaC has divergent roles in vascular responsiveness in conduit and resistant arteries of mice [67]: In carotid arteries, ENaC mediates flow‐dependent vasoconstriction while in conduit arteries ENaC acts as a vasodilator. The underlying reasons for this obviously local differences in ENaC function are unclear yet.

The important and novel finding of this work was to demonstrate the ENaC-dependent mechanosignaling in ApoE/LDLR^-/-^ mice was lost and can thus be seen as an indicator for disturbed mechanosensing and subsequent signaling.

**Fig. 6:**
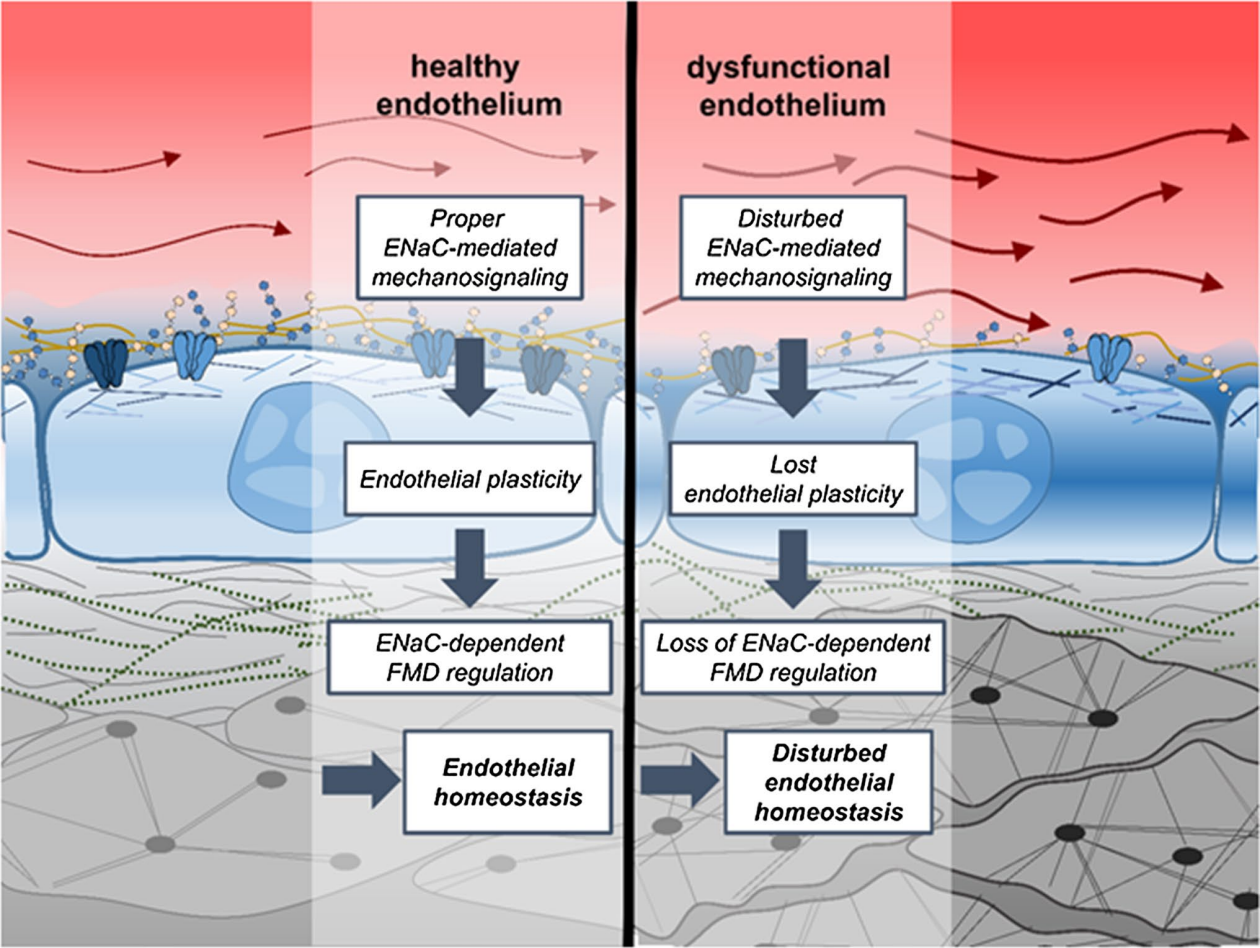
Maladaptive loss of ENaC-dependent regulation of endothelial response to flow contributes to endothelial dysfunction Left side: in a healthy condition, the endothelial cells can flexible adapt to changes in their environment, e.g., different rates of shear stress. This includes an appropriate ENaC-dependent mechanotransduction and the ability of the vessel to react to flow with dilation. Right side: in case of a damaged and atheroprone endothelium, the endothelial cells lost their plasticity. This includes disturbed regulation and mechanotransduction via ENaC, chronic stiffening of the cortex and as a result a loss of normal functional properties of the vessel. It could be debated if such conditions further impair ENaC function, supposing a vicious circle of vascular dysfunction

Altogether, based on these results, we postulate the following scenario: under physiological shear stress conditions (LSS and WT), upon signal transduction via the eGC, ENaC is inserted MR-dependent into the membrane of endothelial cells, where it contributes to proper endothelial function including the ability to regulate endothelium-dependent vasodilation. Under atheroprone conditions (NLSS and ApoE/LDLR^-/-^), in contrast, an ENaC-independent reorganization of the actin cytoskeleton occurs, which stiffens the cortex, endothelium-dependent vasodilation is impaired and ENaC-dependent mechanism regulating endothelium-dependent function is lost (Fig. 6). This is in agreement with data showing that endothelial cells, derived from critically ill patients with an increased pulse wave velocity, have lost the ability to functionally respond to ENaC inhibition [29]. Based on these results, we postulate that malfunction of ENaC mechanosignalling manipulates endothelial nanomechanics which in turn leads to SECS and disturbed vascular homeostasis. This loss of homeostasis is considered to lead to pathological conditions as soon as adaptive physiological mechanisms of ENaC mechanosignalling are replaced by maladaptive mechanisms contributing to endothelial dysfunction.

To conclude, our results suggest that impaired flow-mediated dilation, paralleled by an increased endothelial stiffness, might be related to a disturbed ENaC-glycocalyx-mediated mechanosensing leading to a loss of endothelial homeostasis. This new mechanism contributes to the field of mechanomedicine and provides a novel mechanisms by which ENaC preserves endothelial hemostasis but when this mechanism goes awry and endothelial dysfunction develops represents a potentially useful therapeutic target. The major challenge is to better understand the molecular mechanism underlying the switch from adaptive to maladaptive ENaC-glycocalyx-mediated mechanosensing to preserve endothelial hemostasis and to prevent or reduce cardiovascular pathologies.

## Data Availability

The datasets generated during the current study are available from the corresponding authors on reasonable request.
